# Interstitial Lung Disease in Rheumatoid Arthritis Remains a Challenge for Clinicians

**DOI:** 10.3390/jcm8122038

**Published:** 2019-11-21

**Authors:** Elisabeth Bendstrup, Janne Møller, Sissel Kronborg-White, Thomas Skovhus Prior, Charlotte Hyldgaard

**Affiliations:** 1 Center for Rare Lung Diseases, Department of Respiratory Diseases and Allergy, Aarhus University Hospital, Palle Juul-Jensens Boulevard 99, 8200 Aarhus, Denmark; 2 Diagnostic Centre, University Research Clinic for Innovative Patient Pathways, Silkeborg Regional Hospital, Falkevej 1-3, 8600 Silkeborg, Denmark

**Keywords:** rheumatoid arthritis, interstitial lung disease, usual interstitial pneumonia, non-specific interstitial pneumonia, organizing pneumonia, therapy

## Abstract

Interstitial lung disease (ILD) is a serious complication of rheumatoid arthritis (RA) contributing to significantly increased morbidity and mortality. Other respiratory complications, such as chronic obstructive pulmonary disease and bronchiectasis, are frequent in RA. Infections and drug toxicity are important differential diagnoses and should be considered in the diagnostic work-up of patients with RA presenting with respiratory symptoms. This review provides an overview of the epidemiology and pathogenesis of RA-ILD, the radiological and histopathological characteristics of the disease as well as the current and future treatment options. Currently, there is no available evidence-based therapy for RA-ILD, and immunosuppressants are the mainstay of therapy. Ongoing studies are exploring the role of antifibrotic therapy in patients with progressive fibrotic ILD, which may lead to a new treatment approach for subgroups of patients with RA-ILD.

## 1. Introduction

Rheumatoid arthritis (RA) is a systemic inflammatory disease primarily affecting synovial joints with possible involvement of other organs. It is most often diagnosed in the fourth and fifth decades of life, and women are affected three times as often as men. The lung is a common site of extra-articular disease. Rheumatoid arthritis-associated interstitial lung disease (RA-ILD) is one of the most feared manifestations and causes serious morbidity and increased mortality [1,2,3]. Rheumatoid arthritis is the most common of the connective tissue diseases, and a substantial number of patients with RA will consult general practitioners, rheumatologists, and pulmonologists with respiratory symptoms which, in some cases, may be caused by ILD. Evaluation of respiratory symptoms in patients with RA is challenging because of the many potential causes to be considered including ILD, chronic obstructive pulmonary disease (COPD), bronchiectasis, respiratory infections following immunosuppressive therapies, drug-induced pulmonary toxicity, and ischemic heart disease [4].

The aim of the present review was to provide an overview of epidemiology, diagnostics, treatment, and prognosis in RA-ILD.

## 2. Epidemiology and Risk Factors

Clinically significant RA-ILD is identified in 2% to 10% of patients with RA, but reported estimates vary due to the heterogeneity of RA, genetic susceptibility, and differences in disease definition and diagnostic methods [3,5,6,7,8].

The lifetime risk of developing ILD in patients with RA is reported to be 6%–15% [9,10,11]. Interstitial lung disease may precede the development of articular manifestations. Hyldgaard et al. [5] found that 14% of patients with RA-ILD had been diagnosed with ILD one to five years before the RA diagnosis [3]. The risk of developing ILD increases with prolonged duration of RA. Several risk factors for development of ILD in patients with RA have been identified. The most consistently reported risk factors include older age and male gender [5,11,12], cigarette smoking [12,13], positive anti-cyclic citrullinated peptide antibodies (anti-CCP) or IgM rheumatoid factor [5,12,14], and, in some studies, RA disease activity [5,11]. Smoking is the only preventable risk factor. 

## 3. Pathogenesis

A combination of genetic predisposition and environmental factors seem to be involved in RA-ILD. Restrepo et al. [5] found an increased susceptibility to RA-ILD in smokers with human leukocyte antigen (HLA) DRB1 shared epitopes. This HLA genotype has previously been shown to increase the risk of RA and anti-CCP, especially in smokers [5,15]. Other HLA variants are also associated with RA-ILD [16,17,18].

These HLA variants have a higher binding affinity to citrullinated proteins and an increased tendency to present citrullinated peptides to immune cells. The initial event may take place in mucosal sites [19]. Citrullination is the conversion of the amino acid arginine into the amino acid citrulline. Anti-citrullinated protein antibodies (ACPAs) are synthesized in genetically predisposed individuals during inflammation; these antibodies are found in the majority of patients with RA [20]. The ACPAs are directed against peptides and proteins that have been citrullinated, and smoking induces protein citrullination in the lungs [21]. Thus, the lungs may be the site of the initial immune dysregulation, leading to the development of RA, at least in smokers [19,22,23]. Paulin et al. [24] hypothesized that two potential pathways may explain different patterns of ILD in patients with RA:

(1) The immune response in RA primarily takes place in the synovial tissue in the joints. In some patients, citrullinated peptides may also be produced in the lungs, possibly causing an immune response. Fibroblasts in the lungs are activated and differentiate into myofibroblasts that produce fibrosis. Due to the inflammatory nature of the initial changes, interstitial lung inflammation develops. This process may explain the formation of a predominantly inflammatory disease pattern such as non-specific interstitial pneumonia (NSIP);

(2) In patients with fibrotic ILD, ageing alveolar epithelial cells gain the ability to secrete substances that promote the formation of tissue fibrosis in patients with a genetic susceptibility to RA. The ACPAs synthesized due to the citrullination in the lungs may also affect other tissue showing citrullinated peptides, e.g., synovium. This process may explain the formation of a predominantly fibrotic disease pattern such as usual interstitial pneumonia (UIP) [24,25].

Genetic factors may also play a role in the development of RA-ILD. Juge et al. [26] performed a whole exome sequencing study on patients with RA-ILD and found mutations in familial pulmonary fibrosis-risk genes (*TERT*, *RTEL1*, *PARN,* and *SFTPC*). An association between the *MUC5B* gene variant, a protein coding gene associated with the lubricating and viscoelastic properties of lung mucus, and RA-ILD has been found, similar to findings in idiopathic pulmonary fibrosis and chronic hypersensitivity pneumonitis [8,27].

## 4. Symptoms and Clinical Features 

### 4.1. Symptoms

Symptoms of ILD are indistinguishable from a number of more common lung diseases and include exertional dyspnea, cough, chest pain, and fatigue [28,29,30,31].

A clinical examination may show digital clubbing and/or Velcro-crackles on lung auscultation in patients with fibrotic ILD. Clubbing has been reported in up to 15% of patients with RA-ILD [32]. Bilateral basal crackles have been reported in 72%–100% of patients with RA-ILD. Crackles were also present in patients with RA without ILD but to a smaller extent [23,32]. The variability of the clinical appearance is most likely due to the heterogeneity of the disease and variability in high-resolution computed tomography (HRCT) patterns.

### 4.2. Imaging

Chest X-ray is an insensitive method to detect ILD in patients with RA. Up to 64% of patients with ILD on HRCT will not have evident interstitial changes on a chest X-ray [29,33]. Therefore, HRCT is a mandatory part of the diagnostic work up if ILD is suspected.

The presence of lung abnormalities has been reported in 47%–67% of unselected patients with RA examined by HRCT [31,33,34,35,36,37]; ILD, respiratory disease, and bronchiectasis are common findings. 

**Figure 1:**
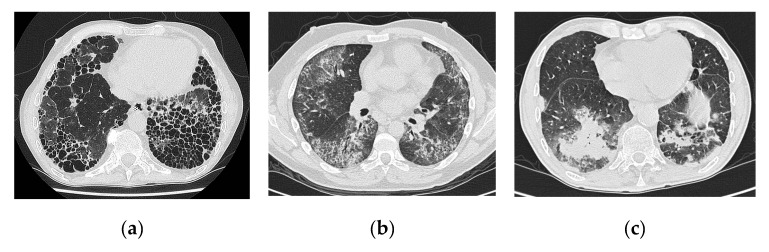
HRCT images showing (**a**) rheumatoid arthritis-associated usual interstitial pneumonia (RA-UIP), (**b**) rheumatoid arthritis-associated non-specific interstitial pneumonia (RA-NSIP), and (**c**) rheumatoid arthritis-associated organizing pneumonia (RA-OP).

Radiological findings in RA-ILD include ground glass-opacities, reticulation, consolidation, honeycombing, and nodules similar to other ILD subtypes [30,33,38,39]. The most common HRCT patterns in RA-ILD are UIP and NSIP, while organizing pneumonia (OP) and bronchiolitis are less common (Figure 1) (Table 1) [8,14,38,39,40,41,42,43,44,45,46,47]. Studies show a fairly good correlation between HRCT and histopathological findings with the least concordance in the diagnosis of UIP and NSIP [39,42,46]. 

Interstitial changes on HRCT often precede symptoms, but interstitial lung abnormalities do not always progress to clinically significant ILD [23,50]. Interstitial lung disease on HRCT has been reported in between 33% and 61% of asymptomatic patients with RA [23,51]. However, uncontrolled arthritis may cause impaired physical activity and may disguise respiratory symptoms.

Lung ultrasonography (LUS) has been a suggested modality to identify ILD by detection of sonographic B-lines. Lung ultrasonography findings have a high sensitivity (89%–97%) but varying specificity (50%–97%) compared to HRCT in patients with RA-ILD [52,53]. However, the etiology of the B lines may be difficult to establish in clinical practice, and differentiation between inflammatory and fibrotic changes is not possible using LUS [54].

### 4.3. Pulmonary Function

Pulmonary function tests (PFTs), including diffusing capacity of the lung for carbon monoxide (DLCO), may detect pulmonary disease. However, the usefulness of PFTs as a screening tool for ILD in RA is limited by the variability within the normal range and by the presence of concomitant emphysema. The results of PFTs among patients with RA-ILD depend on the study cohorts and disease severity.

Abnormal PFT are seen in 45%–65% of patients with RA with or without respiratory symptoms [33,37,55]. The patterns include airway obstruction, restrictive patterns, and impaired DLCO. The prevalence of a restrictive pattern is 5%–25%; impaired DLCO is seen in approximately 20%–45% of patients with RA [55,56,57]. Although a large number of patients have abnormal PFTs, most abnormalities remain clinically insignificant and asymptomatic. There are no screening recommendations for lung disease among patients with RA, and it remains a challenge to decide how to manage minor pulmonary function impairment.

### 4.4. Bronchoscopy with Bronchoalveolar Lavage

Bronchoscopy with bronchoalveolar lavage (BAL) can be used to rule out infection as a cause of pulmonary infiltrates and to assess the profile of the inflammatory cells in the lungs. However, reports of cell counts have been ambiguous. Although increased BAL lymphocyte counts have been found, findings are not consistent [30,58,59]. However, these studies generally included few patients with RA-ILD, and larger studies are needed to determine the prognostic and therapeutic significance of BAL cytological differential counts.

### 4.5. Histopathology

The histopathological patterns in RA-ILD are diverse and all patterns of interstitial pneumonia may occur and even overlap. The classification of the idiopathic interstitial pneumonias [60] distinguishes among a number of specific histopathological patterns which are also seen in RA-ILD. A UIP pattern is characterized by heterogeneity with patchy fibrosis with honeycombing and fibroblast foci alternating with areas of normal lung tissue. Non-specific interstitial pneumonia has a uniform appearance with a thickening of the alveolar septa with varying degrees of inflammation and fibrosis. In respiratory bronchiolitis, desquamative interstitial pneumonia and follicular bronchiolitis, peribronchiolar inflammation, and fibrosis are present, whereas organizing pneumonia is dominated by intra-alveolar plugs of connective tissue. Diffuse alveolar damage is an acute form of lung damage similar to adult respiratory distress syndrome. 

Findings in biopsy studies of RA-ILD are described below (Table 2). Both UIP and NSIP were the most frequently reported patterns in surgical lung biopsies and autopsy material. Rare patterns include eosinophilic pneumonia, lymphocytic interstitial pneumonia, and obliterative bronchiolitis [30,32,42,61,62,63,64].

Rheumatoid arthritis-associated interstitial lung disease may be classified into two different radiological and histopathological phenotypes: (1) fibrotic disease with a UIP pattern and (2) predominantly inflammatory disease with a non-UIP pattern (e.g., NSIP and OP).

Traditionally, histopathology has been regarded as the gold standard in the diagnosis of ILD, but lung biopsies are associated with a significant risk related to the surgical procedure. However, the correlation between radiological and histopathological patterns is high in RA-ILD although studies are small [30,39,65]. Thus, biopsies in the diagnostic work up of RA-ILD should mainly be used in difficult cases with atypical clinical and radiological features. Transbronchial cryobiopsies have emerged as a new diagnostic tool and may be used more often in the future due to the lower associated mortality and high diagnostic yield [66]. 

## 5. Screening for RA-ILD

Screening for respiratory symptoms should be part of the follow up for RA, and patients should be referred for pulmonary evaluation if symptoms occur. Not only ILD, but also COPD, bronchiectasis, and recurrent infections must be identified and treated. There are no studies validating a specific screening program for RA-ILD.

## 6. Differential Diagnosis

In some cases, lung manifestations may precede the diagnosis of RA. Patients with idiopathic interstitial lung disease should be observed prospectively for signs of systemic disease [3,67,68]. Differential diagnostic considerations should also be part of the follow up and not only of the initial diagnostic work up. The evaluation of clinical or subclinical deterioration in patients with RA-ILD is often challenging, and infection and drug-induced pneumonitis are some of the differential diagnosis that should be considered apart from disease progression (Table 3).

However, drug-induced ILD is relatively rare. A large meta-analysis estimated a prevalence of approximately 1% of patients treated with methotrexate, leflunomide, sulfasalazine or TNF-alpha inhibitors [69]. Disease-modifying anti-rheumatic drugs (DMARDs) are widely used in RA with methotrexate (MTX) as the first-line treatment. Methotrexate is also the drug that is most often suspected to cause pulmonary toxicity, mainly within 6–12 months from treatment initiation [70]. The radiologic pattern in MTX-induced pneumonitis typically resembles the pattern of hypersensitivity pneumonitis with diffuse centrilobular nodules and ground glass opacities. Other radiological ILD patterns are uncommon and more likely related to RA itself [68]. A meta-analysis demonstrated increased risk of lung disease, including infections, with MTX compared to other DMARDS and biological agents [70]. All cases of pneumonitis were reported in studies published prior to 2002. In a small study of 64 patients, Gochuico et al. [23] reported an increased risk of progression of preclinical interstitial changes in 21 patients with RA who received MTX compared to other DMARDS [23]. A systematic literature search found 21 prospective studies of MTX monotherapy but identified only 15 cases of pneumonitis among 3463 patients receiving low-dose MTX (0.43%) for up to 36.5 months [71]. Recently, a Mexican study showed improved survival among patients with RA-ILD treated with MTX [72]. Thus, the use of MTX in RA-ILD seems to have a fair long-term safety and may even improve survival; this is contrary to the results of older studies.

Biological agents such as TNF-alpha inhibitors are also associated with pulmonary toxicity [69]. Risk factors for lung injury are reported to be older age, concomitant immunosuppressive therapy, and pre-existing ILD [73].

Infections may complicate the use of all immunosuppressants therapies, and the risk increases with the burden of immunosuppression [74]. The dysfunctional immune system in RA and pre-existing lung disease further increase the risk of infections. In a retrospective study of 181 patients with RA-ILD, pneumonia was the most frequently reported condition (3.9 per 100 person-years) followed by opportunistic infections (1.5 per 100 person-years). The risk increased with prednisolone doses above 10 mg daily with or without DMARDs. Overall, infection rates were 7.4 per 100 person-years [75].

The risks of fungal and mycobacterial infections and reactivation of latent infections are increased by TNF-alpha inhibitors. Latent tuberculosis should be ruled out by interferon–gamma assays before initiation of therapy [76].

Other differential diagnoses should also be considered. Acute exacerbations of RA-ILD occur with a one-year incidence of 2.8% and may mimic severe respiratory infections [77]. Occasionally, environmental allergens may complicate the condition by causing hypersensitivity pneumonitis [78]. Patients with RA have an increased risk of heart disease including congestive heart failure with pulmonary edema [79]. Heart failure may present as ground glass opacities and smooth thickening of the intralobular septa on HRCT, complicating the assessment of lung involvement.

Patients with RA are at increased risk of lymphoma and lung cancer compared to the general population, and the risk is related to immunosuppression and autoimmunity [80]. Malignancy may mimic RA-ILD changes in the lung with OP-like consolidations (lung cancer, lymphoma) or nodular interlobular septal thickening (lymphangitis carcinomatosis). Chronic obstructive pulmonary disease is common among patients with RA [4], and other smoking-related lung diseases such as respiratory bronchiolitis-ILD, desquamative interstitial lung disease, and Langerhans cell histiocytosis may be seen in RA. Pleural effusions, bronchiectasis, and rheumatoid nodules are other pulmonary manifestations of RA that are seldom mistaken for ILD but may occur simultaneously.

## 7. Treatment

New treatment strategies for RA, including early and effective DMARDs, have resulted in reduced disease activity, but the optimal treatment of lung involvement is still a dilemma [81]. So far, no randomized controlled trials in RA-ILD have been conducted.

Corticosteroids, azathioprine, and mycophenolate are the most widely used therapeutic options, with rituximab or TNF-alpha inhibitors as alternatives in patients with refractory disease. Treatment response is often better in RA-ILD with an inflammatory pattern. Fibrotic disease, especially RA-UIP, tends to be less responsive and the course of disease is similar to that of idiopathic pulmonary fibrosis (IPF) [82].

It is a challenge to determine if watchful waiting or treatment initiation is the appropriate strategy for RA-ILD. Treatment is mainly initiated in clinically significant disease when there is evidence of disease progression. Symptom assessment and pulmonary function tests, including DLCO, should be part of the follow-up. Follow-up HRCT should be guided by symptoms and pulmonary function deterioration. The optimal follow-up interval is not known and will depend on disease course and treatment. Follow-up every 3–6 months in stable disease is often used in clinical practice [83].

It is unknown whether treatment can prevent disease progression in patients with subclinical disease.

Current treatment is empiric and essentially based on immunosuppression. Traditionally, corticosteroids are administrated as a daily oral dose or as pulse courses, and it is tapered over months depending on the tolerability and clinical response [84]. The effect of corticosteroids is believed to be limited to the inflammatory subtypes of RA-ILD such as NSIP and OP [65,85]. Other immunosuppressive drugs such as TNF-alfa-inhibitors, methotrexate, azathioprine, mycophenolate mofetil (MMF), and cyclophosphamide are used as maintenance therapy or in corticosteroid resistant cases [84].

In a study from 2013, MMF was shown to stabilize lung function in connective tissue disease-associated ILD (CTD-ILD) including a group of patients with RA (*n* = 18) with progressive decline in forced vital capacity (FVC). The MMF treatment resulted in a trend for FVC increase and was well tolerated [86] which was also reported in previous studies [87,88]. Saketkoo et al. [89] found that MMF improved symptoms and stabilized or improved PFTs in a small mixed CTD cohort that included RA patients. Furthermore, steroids could be tapered.

Treatment of fibrotic CTD-ILD with AZA and MMF resulted in stabilization of lung function in a study from 2016. Mycophenolate mofetil (MMF) seemed to be better tolerated as discontinuation of medication for non-pulmonary side effects was more common in patients on azathioprine (27%) than on mycophenolate (5%). The adverse incident rates were similar in the two groups and did not differ on the basis of HRCT patterns [90]. A retrospective study of 40 RA-ILD patients treated with high-dose glucocorticoids, leflunomide or methotrexate showed improvement in FVC after 4 months [91].

In summary, primarily MMF demonstrates some efficacy with stabilization or minor improvement of lung function combined with relatively low toxicity. Both AZA and MTX seem to have less efficacy. Larger clinical trials are needed to clarify their use in RA-ILD.

The use of cyclophosphamide in combination with methylprednisolone has shown some benefit in rapidly progressive, severe ILD and in RA-ILD with extensive UIP, but evidence is based on small retrospective case series [48,92].

Rituximab, a B-cell-depleting anti-CD20 antibody, was used in 700 patients with RA; 56 had RA-ILD prior to rituximab treatment. A total of 68% of these patients improved or had stable pulmonary function after rituximab [93]. Rituximab was safe and only three patients (0.4%) developed ILD after RTX treatment, which may point towards a protective effect of rituximab [93]. A similar response was seen in smaller case series [94]. Thus, rituximab may be useful in patients with CTD-ILD with severe ILD refractory to other forms of immunosuppression [95].

The use of TNF-alpha inhibitor therapy in RA-ILD is limited to small case series with reports of ILD stabilization [96,97].

Non-pharmacological initiatives should always be considered. As in any chronic lung disease, pulmonary rehabilitation is important, and it has been shown to improve walking distance, quality of life, and dyspnea [98]. Pulmonary physiotherapy, including breathing exercises, may be helpful in managing dyspnea and productive cough.

Influenza and pneumococcal vaccination and optimized hand hygiene should be emphasized as tools to prevent infections (345).

Smoking cessation is essential, and advice should be offered to all smokers with RA-ILD as well as those with only RA. Supplementary oxygen can be beneficial as part of palliative care for patients with hypoxemia.

Lung transplantation should be considered in the most severe and progressive cases. Selection of transplant candidates follows the general recommendations [99]. Extra-pulmonary RA manifestations may compromise the patient’s functional ability, and side effects of immunosuppression may complicate or contraindicate transplantation. Studies have demonstrated similar one-year survival in patients with RA-ILD as in IPF and scleroderma-associated ILD [100,101].

Rheumatoid arthritis-associated interstitial lung disease with a UIP pattern shares radiological and histopathological similarities with IPF, and this raises the question of the role of antifibrotic therapies in the treatment for RA-ILD. A recently published study showed a positive effect of nintedanib on slowing the decline of lung function in a broad range of fibrosing interstitial lung diseases with a progressive phenotype including RA-ILD [102]. Ongoing studies are evaluating another antifibrotic drug, pirfenidone, in RA-ILD and progressive fibrotic ILD [103,104].

## 8. Prognosis

Survival in patients with RA has improved in recent years, but patients with RA-associated ILD still have significantly reduced survival compared to patients with RA alone [3,49,105]. Previous RA-ILD studies report a median survival of three to ten years [11,106,107,108]. More recent studies tend to show longer survival which may be attributed to improved radiological techniques and increased awareness of RA-ILD leading to earlier diagnosis (Table 4).

Previous studies have reported UIP as the predominant radiological pattern and predictor of mortality [14,42]; however, recent studies report a UIP proportion of 40%–54% [40,47]. A recent study showed that radiology-based prediction models in RA-ILD can identify patients with a progressive fibrosis phenotype [112], and that the visual extent of ILD in combination with FVC values, as previously seen in scleroderma [113], predicts outcome in RA-ILD. Morisset et al. [47] showed that the addition of HRCT patterns to the ILD-GAP prediction model did not improve its performance.

Solomon et al. [107] showed that pulmonary physiology independently predicted mortality, but baseline HRCT patterns did not. These findings reflect the heterogeneous and often unpredictable disease behavior in RA-ILD.

## 9. Future Directions for RA-ILD

Rheumatoid arthritis-associated interstitial lung disease remains a challenge for clinicians due to the fact of its heterogeneity and variable course of disease. In recent years, our knowledge of risk factors, pathogenesis, and diagnostic approaches in RA-ILD has increased, but major gaps are still present (Table 5). Expert opinion, case series, and retrospective studies constitute the foundation for treatment recommendations. The first randomized controlled trials are ongoing with results expected in the end of 2019 and 2020, and this may result in the emergence of the first evidence-based treatment options in RA-ILD.

## 10. Conclusions

Rheumatoid arthritis-associated interstitial lung disease is a serious complication of RA contributing to significantly increased morbidity and mortality. Infections, drug toxicity, and other respiratory and cardiac diseases are important differential diagnoses and should be considered in the diagnostic work up of patients with RA. The predominant HRCT and histopathology patterns are UIP or NSIP. There is no evidence-based therapy in RA-ILD. Immunosuppressants remain the mainstay of therapy, but ongoing studies are exploring the role of antifibrotic therapy in patients with progressive fibrotic ILD.

## Figures and Tables

**Table 1:** High-resolution computed tomography (HRCT) patterns in patients with rheumatoid arthritis-associated interstitial lung disease (RA-ILD).

Studies	Total Number of Subjects with ILD (*n* = 1138)	UIP (*n*)	Non-UIP (*n*)	Subtypes of Non-UIP (*n*)					HRCT Not Performed
				NSIP (*n*)	Bronchiolitis (*n*)	OP (*n*)	DAD (*n*)	Other (*n*)	
Tanaka et al. *Radiology* 2004 [39]	63	26	37	19	11	5	-	2	-
Mori et al*. Journal of Rheumatology* 2008 [41]	25	2	23	11	10	2	-		-
Kim et al. *European Respiratory Journal* 2010 [42]	84	20	64	19	-	-	-	45	-
Tsuchiya et al. *European Respiratory Journal* 2011 [45]	102	57	26	16	-	5	5	-	19
Kelly et al. *Annals of Rheumatology Dis* 2014 [48]	231	150	81	55	-	12	-	14	-
Assayag et al. *Radiology* 2014 [46]	69	38 *	31 **	-	-	-	-	-	-
Yunt et al*. Respiratory Medicine* 2017 [43]	195	123	72	35	28	4	-	5	-
Zhang et al. *Annals of Rheumatic Diseases* 2017 [49]	237	44	193	137	-	-	-	56	-
Juge et al. *New England Journal of Medicine* 2018 [8]	620	207 *	298 **	-	-	-	-	-	-
Zamora-Legoff et al. *Rheumatology* 2017 [40]	181	98	77	73	-	4	-	-	-
Morisset et al. *Respiratory Medicine* 2017 [47]	309	125 *	184 **	-	-	-	-	-	-
Nurmi et al. *Respiratory Medicine* 2018 [44]	60	36	24	8	-	7	1	8	-
% of total	100%	43%	51%	17%	2%	2%	0%	6%	1%

* Both definite and possible UIP. ** Inconsistent with UIP. UIP: usual interstitial pneumonia, NSIP: non-specific interstitial pneumonia, OP: organizing pneumonia, DAD: diffuse alveolar damage.

**Table 2:** Histopathological patterns from surgical lung biopsy and autopsy materials in patients with RA-ILD. UIP: usual interstitial pneumonia, NSIP: non-specific interstitial pneumonia, OP: organizing pneumonia, DAD: diffuse alveolar damage.

	Number	UIP	NSIP	Bronchiolitis	OP	DAD	Unclassifiable
Tansey et al. *Histopathology* 2004 [61]	16	2	7	7	-	-	-
Lee et al. *Chest* 2005 [30]	18	10	6	2	-	-	-
Yoshinouchi et al. *Rheumatology. International.* 2005 [62]	16	7	7	-	-	-	2
Kim et al*. Chest* 2009 [65]	14	10	3	-	-	-	1
Nakamura et al*. Respiratory Medicine* 2012 [32]	54	15	16	17	4	-	2
Yousem et al. *American Review of Respiratory Disease* 1985 [63]	19	5	5	1	6	2	-
Tanaka et al. *Radiology* 2004 [39]	17	2	10	2	2	1	-
*N* (% of total)	161 (100%)	57 (35%)	54 (34%)	29 (18%)	13 (8%)	3 (2%)	5 (3%)

**Table 3:** Differential diagnosis of RA-ILD.

Drug-induced lung toxicities *
Infections **
Bronchiectasis
COPD
Congestive heart failure
Pleural effusions
Malignancy (lung cancer, lymphoma)
Acute exacerbations in RA-ILD
Smoking-related parenchymal lung diseases †
Rheumatoid nodules

Disease-modifying anti-rheumatic drugs (DMARD) and biologic therapy. ** Pneumonia and opportunistic infections. **†** Respiratory bronchiolitis-interstitial lung disease (RB-ILD), Langerhans cell histiocytosis. COPD: chronic obstructive pulmonary disease.

**Table 4:** Reported HRCT patterns and mortality in RA-ILD studies published between 2009 and 2019.

Authors	Institution	Study Period	RA-ILD Cohort Size	HRCT Patterns	Survival
Bongartz et al. *Arthritis & Rheumatology* 2010 [11]	Population-based incidence cohort of RA patients in Rochester, USA	1955–1995	46	Not reported	Median survival 2.6 years
Kim et al. *European Respiratory Journal* 2010 [42]	Two referral centers, UCSF and Mayo Clinic, USA	2001–2008	82	24% UIP 23% NSIP 51% indeterminate 2% other	Median survival UIP 3.2 years Not UIP 6.6 years
Koduri et al. *Rheumatology* 2010 [106]	ERAS Inception cohort of RA patients from nine rheumatology centers in the UK	1986–1998	52	Not reported	Median survival 3 years
Tsuchiya et al. *European Respiratory Journal* 2011 [45]	Respiratory center, Japan	1996–2006	102	56% UIP 16% NSIP 5% OP 5% DAD 19% combined	Five-year survival UIP 36.6% NSIP 98.3% OP 60.0% DAD 20%
Nakamura et al. *Respiratory Medicine* 2012 [32]	Referral center, Japan	1980–2009	54	Not reported	Ten-year survival 76.6% (UIP 52.5%, NSIP 84.3%)
Solomon et al. *Respiratory Medicine* 2013 [108]	Two referral centers. National Jewish and Mayo Clinic, USA	1977–1999	48	Not reported	Median survival 45 months
Assayag et al. *Radiology* 2014 [46]	Three referral centers, USA and Korea	1997–2011	69	29% definite UIP 26% possible UIP 45% inconsistent with UIP	Not reported
Kelly et al. *Rheumatology* 2014 [14]	BRILL network, Rheumatology centers in the UK	1987–2012	230	65% UIP 24% NSIP OP 6% Overlap syndromes 6%	Not reported
Nurmi et al. *BMC Pulmonary Medicine* 2016 [109]	Referral center, Kuopio, Finland	2000–2014	59	59% UIP 14% NSIP 12% OP 15% other	Median survival UIP 92 months Not UIP 137 months
Solomon et al. *European Respiratory Journal* 2016 [107]	Referral center, National Jewish, USA	1995–2013	137	79% UIP 21% NSIP	Median survival 10.35 years UIP 10.2 years NSIP 13.6 years
Morisset et al. *Respiratory Medicine* 2017 [47]	Four referral centers, USA, Korea, Italy	NA	309	24% UIP 16% possible UIP 60% inconsistent with UIP	Three-year mortality: GAP stage 1 1.9% GAP stage 2 17.6% GAP stage 3 50.3%
Nurmi et al. *BMC Pulmonary Medicine* 2017 [110]	Referral center, Kuopio, Finland	2000–2014	59	Reported in a previous study	Median survival GAP stage 1, 152 months, GAP stage 2, 61 months Three-year mortality GAP stage 1, 17.6% GAP stage 2, 27.3%
Rojas-Serrano et al. *Clinical Rheumatology* 2017 [72]	Mexico	2004–2015	78	26% UIP 36% NSIP 19% LIP 6% OP 36% overlap	Median survival 5.8 years
Yunt et al. *Respiratory Medicine* 2017 [43]	Referral center, National Jewish, USA	1995–2014	158	63% definite UIP 15% possible UIP 22% NSIP	Median survival Definite UIP 2.77 years Possible UIP 6.14 years NSIP incalculable
Zamora-Legoff et al. *Rheumatology* 2017 [40]	Referral center, Mayo Clinic, USA	1998–2014	181	54% UIP 40% NSIP 6% OP	Five-year survival 59.7%, no difference among HRCT groups
Zhang et al. *Clinical Rheumatology* 2017 [38]	China	2008–2013	237	18.6% UIP 57.8% NSIP	Not reported
Juge et al. *New England Journal of Medicine* 2018 [8]	France, China, Greece, Japan, Mexico, the Netherlands, and USA	NA	620	41% UIP/possible UIP 59% inconsistent with UIP	Not reported
Nurmi et al. *Respiratory Medicine* 2018 [44]	Referral center, Kuopio, Finland	2000–2014	60	Reported in a previous study	Not reported
Sparks et al. *Arthritis & Rheumatology* 2019 [111]	BRASS cohort, USA	2003–2016	85	49% fibrotic NSIP 32% cellular NSIP 19% UIP/AIP/DAD	37.6% died during follow-up

GAP: Gender, age and physiology score.

**Table 5:** Future research needs in RA-ILD.

Deeper insights into the pathogenesis of RA-ILD and its relationship to the pathogenesis of other ILDs Better understanding of inflammatory versus fibrotic phenotypes in RA-ILD Possible benefits of computerized automatized CT Potential benefits of histopathologic evaluation of the degree of inflammation and fibrosis Potential benefits of combination therapy with anti-inflammatory and antifibrotic drugs Deeper insight into genetic phenotyping and tailoring of therapy Potential benefits of improved smoking cessation programs for all RA patients
